# Functional outcome of contralateral C7 nerve transfer combined with free functional gracilis transplantation to repair total brachial plexus avulsion: a report of thirty-nine cases

**DOI:** 10.1007/s00264-021-05108-z

**Published:** 2022-02-03

**Authors:** Jianping Chen, Bengang Qin, Honggang Wang, Jintao Fang, Jiantao Yang, Liqiang Gu

**Affiliations:** Department of Orthopedic Trauma and Microsurgery, the First Affiliated Hospital of Sun Yat-Sen University, Guangzhou, 510008 China

**Keywords:** Contralateral C7 nerve root transfer, Free functional gracilis transplantation, Total brachial plexus avulsion, Function reconstruction, Protective sensibility

## Abstract

**Purpose:**

Treatment of total brachial plexus avulsion (TBPA) is a challenge in the clinic, especially the restoration of hand function. The current main surgical order is from proximal to distal joints. The purpose of this study was to demonstrate the outcomes of “distal to proximal” surgical method.

**Methods:**

Thirty-nine patients underwent contralateral C7 (CC7) nerve transfer to directly repair the lower trunk (CC7-LT) and phrenic nerve transfer to the suprascapular nerve (PN-SSN) during the first stage, followed by free functional gracilis transplantation (FFGT) for elbow flexion and finger extension. Muscle strength of upper limb, degree of shoulder abduction and elbow flexion, and Semmes–Weinstein monofilament test and static two-point discrimination of the hand were examined according to the modified British Medical Research Council (mBMRC) scoring system.

**Results:**

The results showed that motor recovery reached a level of M3 + or greater in 66.7% of patients for shoulder abduction, 87.2% of patients for elbow flexion, 48.7% of patients for finger extension, and 25.6% of patients for finger flexion. The mean shoulder abduction angle was 45.5° (range 0–90°), and the average elbow flexion angle was 107.2° (range 0–142°), with 2.5 kg average flexion strength (range 0.5–5 kg). In addition, protective sensibility (≥ S2) was found to be achieved in 71.8% of patients.

**Conclusion:**

In reconstruction of TBPA, CC7 transfer combined with free functional gracilis transplantation is an available treatment method. It could help patients regain shoulder joint stability and the function of elbow flexion and finger extension and, more importantly, provide finger sensation and partial finger flexion function. However, the pick-up function was unsatisfied, which needed additional surgery.

## Introduction

Traumatic total brachial plexus avulsion (TBPA) is predominantly present in young adults and results in the complete function loss of the upper extremity. Treatment of TBPA is a challenge because there are limited available donor nerves to control the multiple functions that are desirable for the shoulder, elbow, wrist, and hand, and only extraplexal donors can be used [1].

To date, nerve transfer alone or combined with free functional gracilis transplantation (FFGT) is the main options for this irreparable injury. Nerve transfer allows return of some function, but the overall recovery of hand function remains poor [2]. However, FFGT can be used to obtain hand function. In recent years, nerve transfer combined with FFGT or double FFGT was used to restore upper extremity function, especially to improve the hand function[3, 4].

The current surgical order is to restore the elbow flexion and shoulder abduction and followed by wrist and hand function (“proximal to distal”) [2, 3, 5]. Based on the clinical research, the shoulder and elbow function of the affected limb can be significantly improved [6], but the results of hand function are still poor because of the long distance from the site of injury, the slow rate of nerve regeneration, and muscle denervation [7, 8]. To improve the results of hand function in the early stage, our team (Professor L.Q. Gu) described a “distal to proximal” treatment strategy. In the first stage, we performed CC7-LT to restore the protective sensibility of the hand and finger flexion and PN-SSN to restore shoulder abduction. In the second stage, FFGT was used to reconstruct the elbow flexion and finger extension.

The goal of our study was to evaluate the functional outcomes of CC7-LT and PN-SSN combined with FFGT to repair TBPA.

## Patients and methods

This study was approved by the Ethics Committee of the First Affiliated Hospital of Sun Yat-sen University, China (Application ID: [2019] 397) on November 12, 2019. From January 2006 to December 2016, 43 patients suffering from TBPA, including those with PN palsy (3 patients), underwent subsequent surgery at our institution. All patients were diagnosed by history, physical examination, electrophysiological study, and MRI and confirmed by subsequent intra-operative exploration and neurophysiological investigation. Inclusion criteria were (1) TPBA and (2) follow-up at least 24 months, and (3) CC7-LT combined with FFGT was the main reconstruction method. Exclusion criteria were (1) diabetes; (2) fracture in the affected upper extremity (1 case); (3) spinal cord injury; (4) brain injury (1 case); (5) distal amputation of injured limb; and (6) refusal to participate. Two patients were lost to follow-up. Finally, 39 patients qualified for this study.

Pre-operative chest radiography and pulmonary function tests were conducted to exclude any pulmonary disease and permit subsequent PN transfer. CT angiography was undertaken to exclude major vascular injury and assess the patency of suitable recipient vessels for FFGT.

### Surgical techniques

**Fig. 1:**
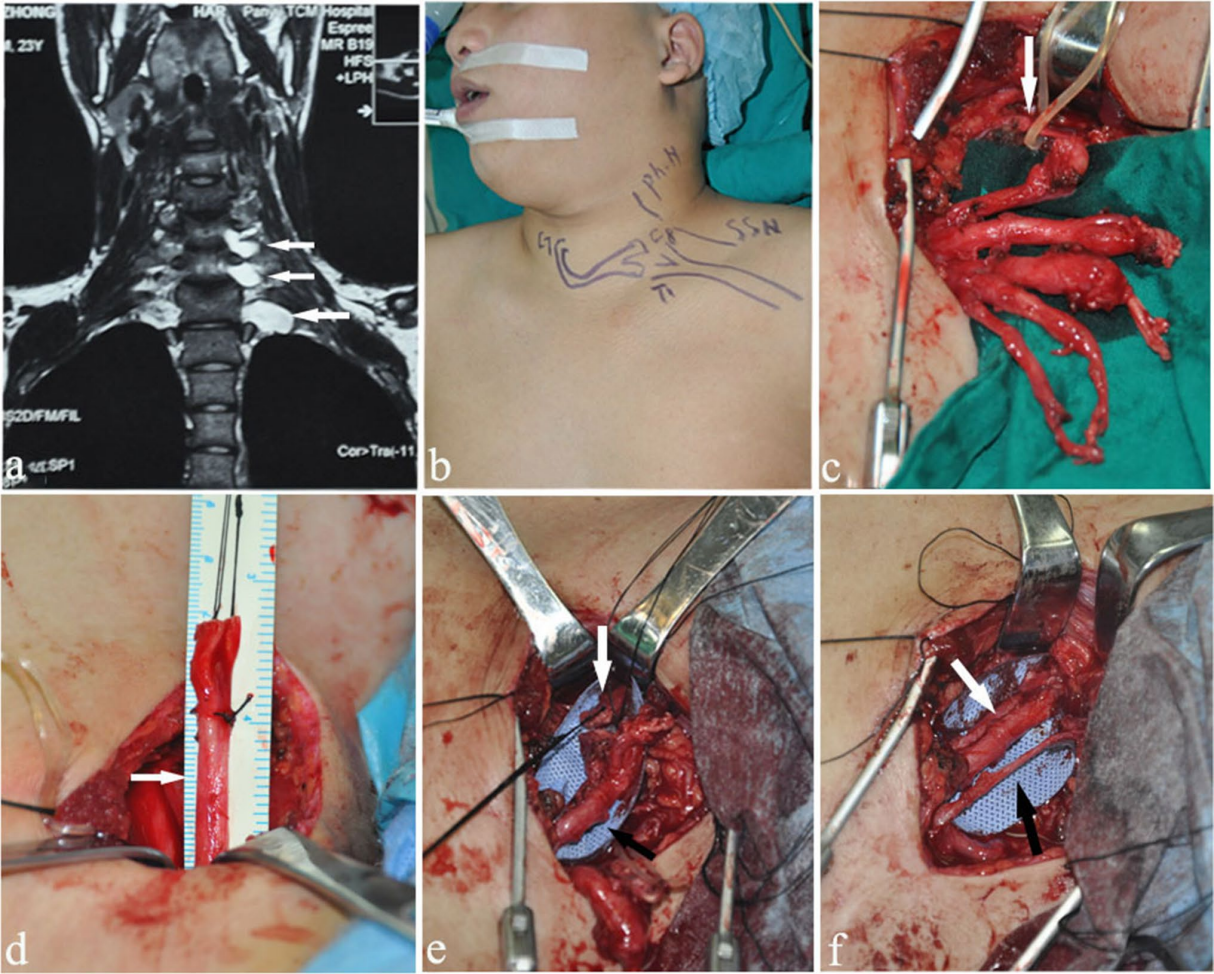
Intra-operative pictures of the first stage. **a** Preoperative MRI of the brachial plexus suggested C5-T1 nerve root avulsion with pseudomeningocele formation (white arrow). **b** Surgical design schematics. **c** Intra-operative photograph showed the C5-T1 nerve root avulsion and the PN (white arrow). **d** The CC7 (white arrow) was identified, transected and measured. **e** There were no gaps between the CC7 (white arrow) and C8T1 (black arrow). **f** The CC7 was directly coapted to C8T1 (white arrow), and the SSN was simultaneously sutured to the PN (black arrow)

All surgical procedures were performed by the same surgeon (L.Q. Gu). In the first stage, details of the surgical techniques have been previously described by Li et al. [9]. The brachial plexus was explored, and the recipient nerves, such as the SSN, lower trunk, or C8T1 nerves, were inspected and prepared. After the CC7 harvest, it was passed to the injured side through the prespinal route and directly coapted to the lower trunk or C8T1. At the same time, the ipsilateral PN was found on the anterior surface of the scalenus anticus muscle and confirmed by electrical stimulation. The PN (36 patients) was transected as distally as possible and then was directly sutured to SSN using 8–0 Prolene sutures (Fig. 1).

**Fig. 2:**
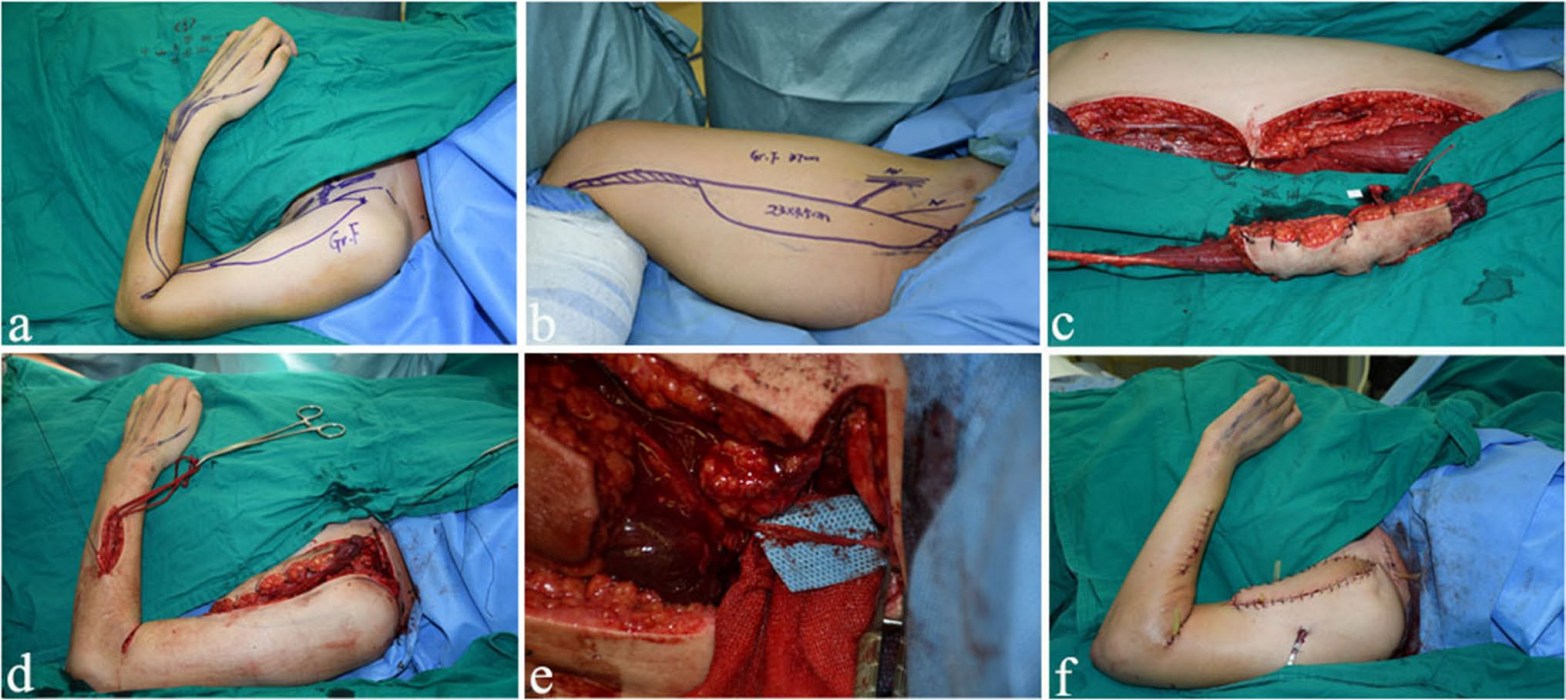
Intra-operative pictures of the second stage. **a** Surgical design schematics for the recipient site before operation. **b** Surgical design schematics for the donor site before operation. **c** The maximum length of the gracilis muscle was dissected from the thigh. **d** The gracilis was inserted subcutaneously into the anteromedial aspect of the arm. **e** The nerve of the gracilis was anastomosed to the SAN. **f** The gracilis muscle maintains a certain tension. A tube was used for drainage after surgery

Timing of the second surgery was determined by monthly follow-ups and measurements. Gracilis muscle harvest was done using the technique described by Yang et al. [10]. In this technique, not only the fascia of the gracilis was preserved but also the fascia of the adductor longus and adductor magnus covered the gracilis. Two teams did the surgery simultaneously: One team isolated the donor vessels, the spinal accessory nerve (SAN), and prepared the proximal and distal attachments of the transferred gracilis. The other team harvested the gracilis muscle. Then, the gracilis muscle was proximally sutured to the acromion or the lateral aspect of the clavicle and distally to the extensor digitorum communis in the forearm. This free functional muscle was perfused by the brachial artery, axillary artery, and subclavian artery with T-shaped anastomosis and refluxed by the comitant vein, and it was innervated by the SAN (Fig. 2).

The additional operation depended on whether there were residual roots in the exploration of brachial plexus, the functional recovery of patients, and their wishes. Additional surgery included C5-C6 stump to repair the upper trunk, wrist fusion, thumb reconstruction, tendon transfer, and second gracilis transfer to reconstruct fingers flexion. The second gracilis muscle was placed on the medial aspect of the arm. It was attached proximally to the tendon of the lesser tuberosity of humerus and routed under the lacertus fibrosus, and distally woven into flexor pollicis longus and flexor digitorum profundus. The recipient nerves were the fourth and fifth ICN nerves. The recipient vessels were brachial artery and vein.

All patients were subdivided into group A and group B according to whether they received additional surgery or not. Group A underwent CC7-LT and PN-SSN and FFGT. Group B underwent the same surgery as group A, as well as additional surgery.

### Post-operative management

The patients were immobilized by wearing a head-shoulder custom cast for six weeks after nerve reconstruction. After removing the cast, the patient was sent to the rehabilitation department for training. In the second stage, the patient was also required to wear a cast with anteflexion and adduction of the shoulder, 90° flexion of the elbow, and finger extension for six weeks. Flap monitoring was performed every hour for the first 48 hours, with a flap colour evaluation and temperature changes using an infrared temperature monitor as parameters [11]. The patient stayed in bed for ten days until the flap survived, and was advised to perform rehabilitation exercises, undergo electrical stimulation therapy, and take neurotrophic drugs after the cast was removed.

### Clinical evaluation

Assessments for this study included clinical measurements of motor and sensory functional recovery. Post-operative functional video assessment was also recorded. Disabilities of the arm, shoulder, and hand (DASH) were scored to assess the function of the injured side [12]. A numeric rating scale (NRS) was used to evaluate pain [13].

### Motor function assessment

The mBMRC grading system [14], with intermediate grades of (-) and ( +), was used for the motor assessment as follows: Poor, M0 to M2; fair, M2 + to M3; good, M3 + or M4 − ; and excellent, M4 to M5 − . Muscle strength of the injured extremity was measured compared to the normal side. Kilogram lifting was used to assess elbow flexion strength in an anatomically neutral position [15]. When the finger flexion and extension strength reached M3, the pick-up function was measured using a small round object.

### Sensory function assessment

The sensory of the hand and fingers was assessed by the Semmese-Weinstein monofilament test [16] and static two-point discrimination (S2PD) [17] and graded according to the mBMRC system. The protective sensibility was graded in S2.

### Statistical analysis

Descriptive data were expressed as frequencies, and continuous data were presented as the mean and standard deviation or as the mean with a range. Student’s unpaired *t* test was used for the analysis between groups A and B. A value of < 0.05 was considered statistically significant.

## Results

The demographic characteristics and functional results of the patients are summarized in Tables 1 and 2.

**Table 1:** Demographic characteristics and functional results of the 39 patients

Factor	Value	Percentage/mean ± SD
Male, no	37	94.8%
Female, no	2	5.2%
Left side, no	26	66.7%
Right side, no	13	33.3%
Cause of injury, no		
Car collision	5	12.8%
Motorcycle collision	21	53.8%
Bicycle collision	1	2.6%
Weight dropping on the shoulder	2	5.2%
Dropping from a height	7	17.9%
Traction injury of upper limb	3	7.7%
Age of injury, years	16–49	26.7 ± 8.7
Interval between injury and op, days	15–210	71.7 ± 49.4
Interval between two stages, days	104–840	315.8 ± 180.0
Gracilis first construction, days	120–330	192.5 ± 49.5
Follow-up period, mos	28–169	97 ± 38.3
Shoulder abduction (°)	0–90	45.5 ± 29.0
Elbow flexion (°)	0–142	107.2 ± 30.1
Elbow flexion strength (Kg)	0–5	2.5 ± 1.2
DASH score	20–52.5	32.5 ± 7.1
NR score	0–8	4.2 ± 1.9

**Table 2:** Postoperative results of muscle strength

	Shoulder abduction		Gracilis		Wrist flexors		Wrist extensors		Thumb flexors		Thumb extensors		2–4th finger flexors		2-4th Finger Extensors	
Results	No. of patients	%	No. of patients	%	No. of patients	%	No. of patients	%	No. of patients	%	No. of patients	%	No. of patients	%	No. of Patients	%
Poor (M0 to M2)	5	12.8	2	5.1	32	82.0	20	51.3	30	76.9	23	59.0	21	53.9	11	28.2
Fair (M2 + to M3)	8	20.5	3	7.7	5	12.8	8	20.5	8	20.5	10	25.6	8	20.5	9	23.1
Good (M3 + or M4-)	4	10.3	5	12.8	1	2.6	8	20.5	1	2.6	6	15.4	8	20.5	16	41.0
Excellent (M4 to M5-)	22	56.4	29	74.4	1	2.6	3	7.7	0	0.0	0	0.0	2	5.1	3	7.7

### Shoulder function

PN and proximal stumps were used to restore the shoulder function. At the final follow-up, 35 patients (89.7%) recovered shoulder abduction function, with an average angle of 45.5° (range 0–90°); 66.7% of patients showed good and excellent shoulder abduction strength.

### Elbow function

Thirty-seven patients had successful reconstruction of elbow flexion; no useful elbow flexion was seen in one patient. The average elbow flexion was 107.2° (range 0–142°); 87.0% of patients had a useful result of M3 + or better, with 2.5 kg average flexion strength (range 0.5–5 kg).

### Hand function

**Fig. 3:**
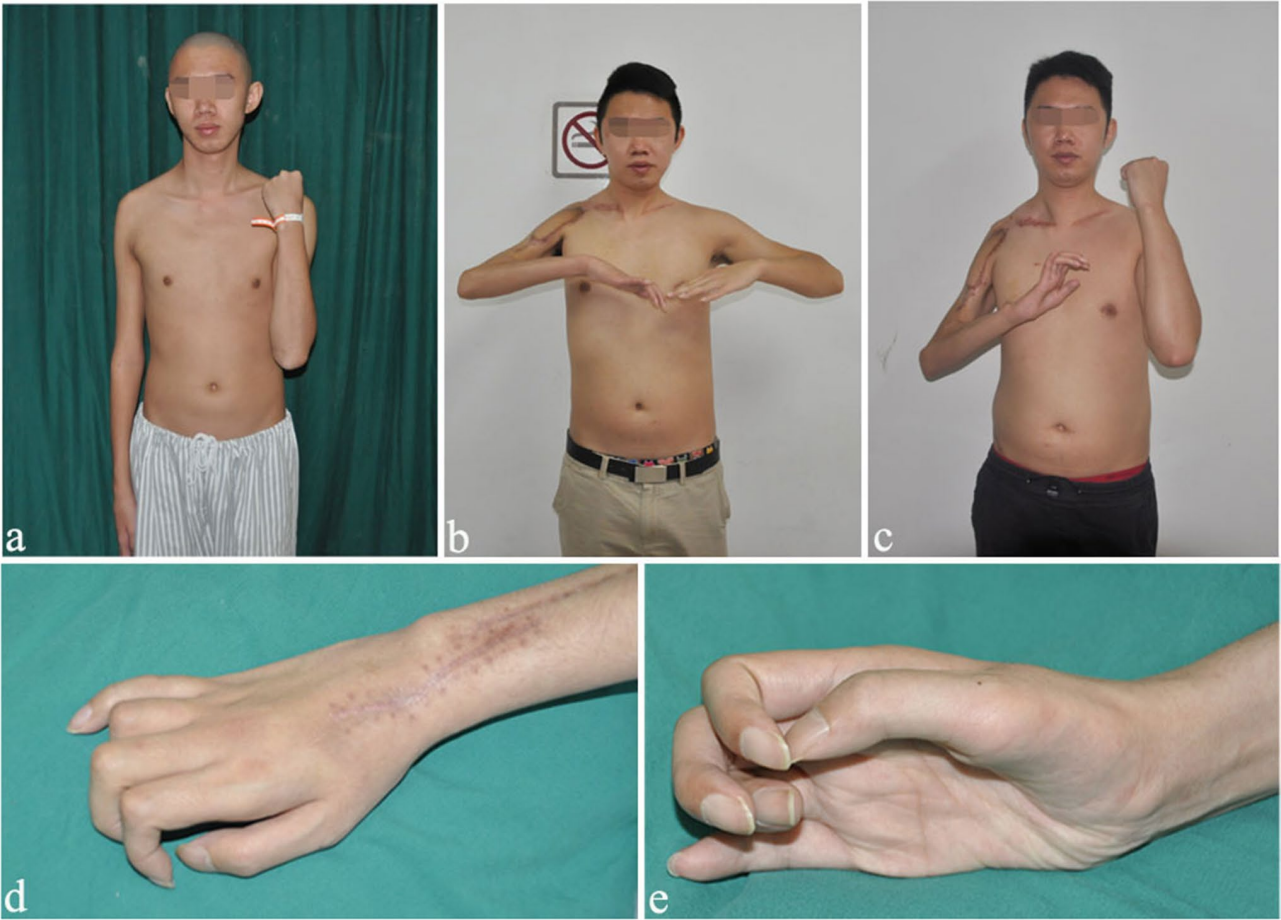
Case 1: pick-up gesture of the fingers. An 18-year-old man sustained TBPA caused by a motorcycle collision. He underwent three operations: CC7 to LT and PN to SSN, FFGT, and wrist fusion. At the 67-month follow-up, he had regained excellent function of shoulder abduction and elbow flexion. Most importantly, he recovered the active pick-up function. **a** Pre-operative view of the right upper limb, which lost the motion and sensory function. **b** Shoulder abduction and elbow flexion before wrist fusion. **c** Elbow flexion after wrist fusion. **d** and **e** Pick-up gesture of the fingers

**Fig. 4:**
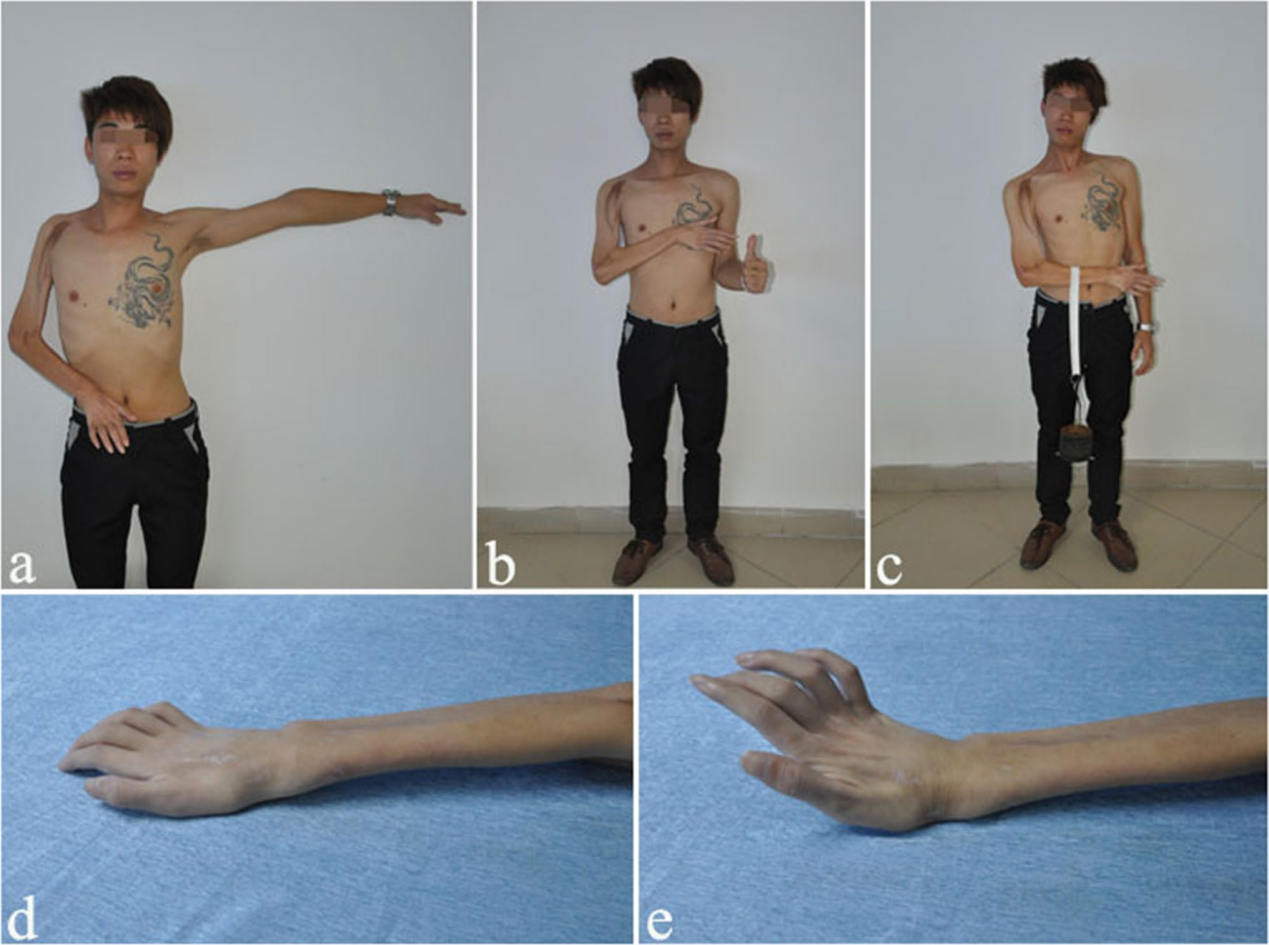
Case 2: hyperextension of the fingers. A 22-year-old man sustained TBPA and underwent only two operations: CC7 to LT and PN to SSN and FFGT. At the 102-month follow-up, he had regained good muscle strength of elbow flexion and finger extension. His metacarpophalangeal joint showed hyperextension, because the finger extension strength (M4-) caused by the gracilis was stronger than the finger flexion strength (M2) restored by the CC7 transfer. **a** Shoulder abduction. **b** and **c** Excellent elbow flexion with a strength of 4 kg. **d** and **e** Wrist and finger extension

**Fig. 5:**
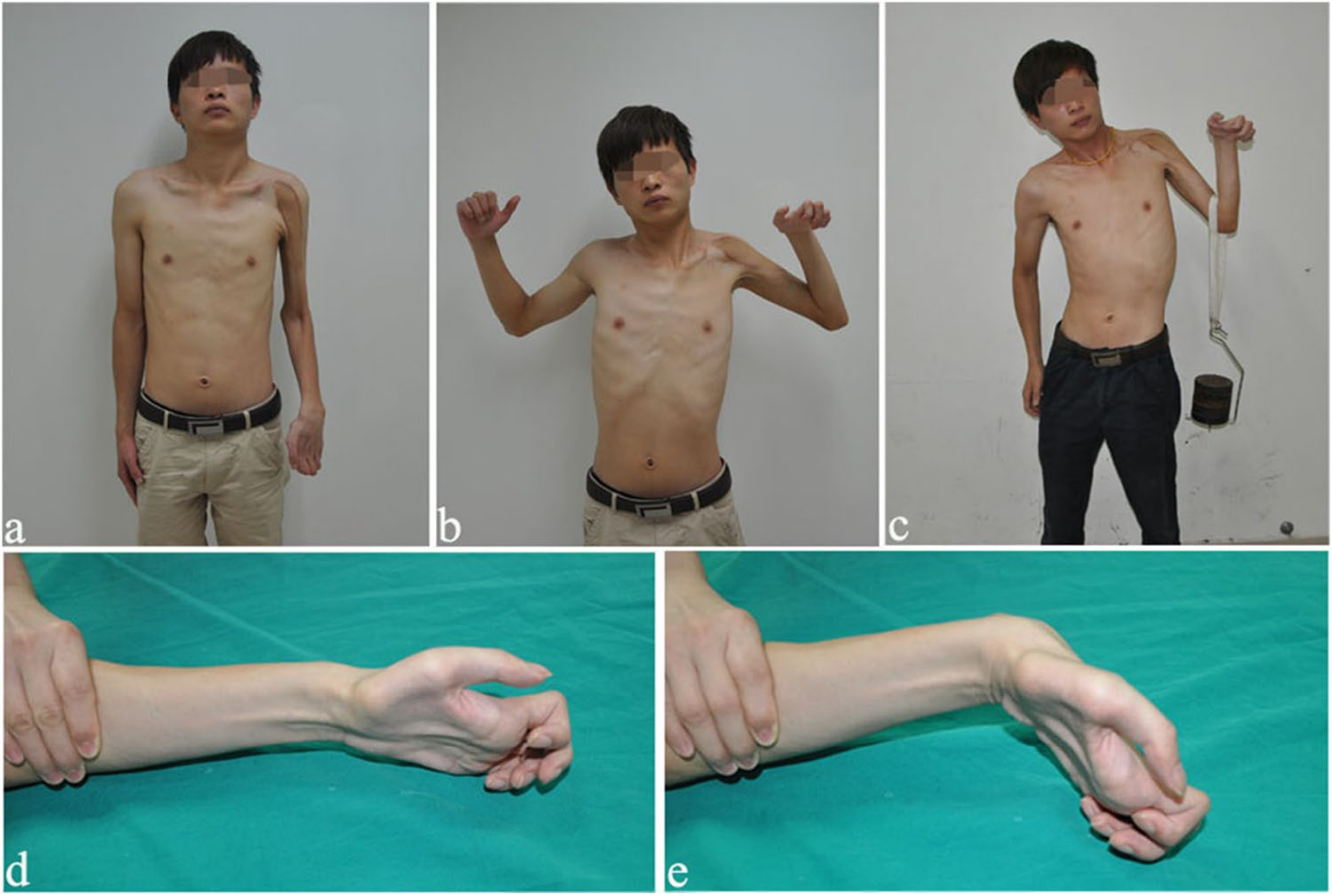
Case 3: hyperflexion of the fingers. A 21-year-old man with TBPA underwent a two-stage operation: CC7 to LT and PN to SSN and FFGT. At the 100-month follow-up, he had regained excellent function of elbow flexion and finger flexion. His fingers showed hyperflexion, because the finger flexion strength (M4) restored by CC7 was stronger than the finger extension strength (M2) caused by the gracilis. **a** Front view after the operation. **b** Shoulder abduction and elbow flexion. **c** Elbow flexion with a strength of 5 kg. **d** and **e** Wrist and finger flexion

The finger strength is summarized in Table 2. 46.1% of patients achieved ≥ M2 + for 2nd–4th finger flexion, 71.8% of patients achieved ≥ M2 + for 2nd-4th finger extension, and nine patients achieved ≥ M3 for finger flexion and extension; only 4 patients regained the pick-up function. All patients showed three gestures as follows: (1) nine patients showed the pick-up gesture, which is good for picking up light things (Fig. 3). (2) Twenty patients showed hyperextension of metacarpophalangeal joints (Fig. 4). (3) Ten patients showed a flexion deformity of the middle and distal interphalangeal joints (Fig. 5).

The patients exhibited sensation of the hand and fingers at 14–18 months after operation. Twenty-eight patients (71.8%) could sense the Semmes–Weinstein monofilament test. Three patients had 4.31, five patients had 4.56, seven patients had 5.07, and 13 patients had 6.65 feeling on monofilament test. Fifteen patients developed an average S2PD sense about 13.5 mm (range 10–19). According to the mBMRC system, 15 patients (38.5%) achieved S3 sensory recovery, 13 patients (33.3%) S2 recovery, and seven patients (18.0%) S1 recovery. There was no sensory recovery found in four patients (10.2%), but their sensation could be located in the contralateral hand.

The percentages of no pain, mild, moderate, and severe pain were 5.1%, 28.2%, 59.0%, and 7.7%, respectively. The average post-operative DASH score was 32.5 ± 7.1 (range 20–52.5).

### Additional surgery

Thirteen patients accepted additional surgery in group B. The proximal nerve stumps of seven patients were available in the first stage. Three case of C5-C6 stump were used to repair the upper trunk with a nerve graft, while other four patients had a direct repair. After the two-stage operation, nine patients accepted further surgery to reconstruct the hand function: two wrist fusions, one wrist fusion and tendon transfer, and six secondary gracilis transfers to provide finger flexion. The shoulder abduction angle in group B was significantly better than that in group A (P = 0.007), and the DASH score in group B was lower than that in group A (P = 0.044). There were no significant differences in the pain score and elbow flexion degree between the two groups (Table 3).

**Table 3:** Comparison of postoperative function and quality of life between group A and group B

Outcome (percentage/mean ± SD)	Group A	Group B	Unpaired *t* test (*P*)
Patients number	26	13	
Shoulder abduction (°)	36.8 ± 5.6	62.8 ± 6.0	0.007
Elbow flexion (°)	105.5 ± 5.4	106.0 ± 9.6	0.278
Finger flexion (> M3)	30.8% (8/26)	76.9% (10/13)	0.007
DASH score	34.1 ± 1.5	29.3 ± 1.4	0.044
NR score	4.2 ± 0.4	4.2 ± 0.4	> 0.999

### Complications

There were some complications in donor side after CC7 transfer. Twenty-one patients had numbness in the index finger, middle finger, and thumb; this symptom recovered three months later. Ten patients reported elbow extension weakness, but they recovered to normal six months later. Complications in the second stage included venous crisis in two cases (one case survived, the other one removed due to flap necrosis) and one case of thigh haematoma found in the donor area one month later.

## Discussion

Nowadays, most authors reported that the philosophy for TPBA reconstruction was from proximal to distal joints [2, 3, 5]. They focused on the recovery of limb motor function while ignoring hand sensation. Several studies have confirmed that satisfactory results can be achieved by restoring shoulder and elbow function, but reconstruction of hand function is very challenging. In addition, the injured hand may be further damaged without sensory recovery, particularly by burns in this contact zone [18]. Based on these, our team tries to restore protective sensibility and finger flexion first, followed by the restoration of elbow flexion and finger extension.

Sensation with or without motor function in the hand and shoulder stability has been the usual aims of nerve reconstruction. Since Gu et al. reported the use of CC7 for TBPA, this procedure had been proved to restore the sensory and motor function of the hand [19]. There were several methods for restoring hand function via a CC7 transfer to the lower trunk or the medial nerve with vascularized ulnar nerve bridging. However, related study demonstrated that CC7 transfer to lower trunk was more effective than to the median nerve in finger flexion [20, 21]. The finger flexion results are varied from one centre to another, but the sensory recovery significantly improves. In the literature, the results of finger flexion by CC7 transfer to the median nerve grade ≥ M3 are approximately 29–34% [22–24]. A systematic review of CC7 transfer to the median nerve showed that 48% of patients achieved ≥ M3 wrist flexion, 42% achieved ≥ M3 finger flexion, and 56% achieved ≥ S3 sensory recovery in the median nerve territories [25]. Bhatia et al. [21] compared two methods for CC7 transfer to the lower trunk: direct repair and nerve graft, the results showed that the finger flexion function of nerve graft group appeared at 16–18 months, a three to six month delay when compared with the direct repair group. In our series, all patients used CC7 transfer to directly repair the lower trunk or C8T1, avoiding the nerve graft. At the final follow-up, 71.8% of patients had a recovery of protective sensibility, while 46.1% of patients achieved grade ≥ M3 finger flexion.

As we know, shoulder stabilization, restoration of abduction, and external rotation are important as more distal functions will be affected by the condition of the shoulder. To achieve this, various techniques have been proposed and used, such as nerve reconstruction in early stages using intra- or extraplexus donors [26] and secondary procedures including arthrodesis, tendon transfers, and muscle transfers [27–29]. One study showed that when 30° of shoulder abduction was achieved, shoulder subluxation was usually corrected [30]. In this series, we used PN to SSN and C5-C6 stumps to upper trunk to reconstruct shoulder function. None of the patients performed shoulder fusion because it yielded a poor range of motion. Our results showed that 27 patients (69.2%) had a shoulder abduction angle greater than or equal to 30°, and 66.7% of patients’ strength of shoulder abduction showed good and excellent results. Post-operative muscle atrophy was improved, and shoulder subluxation disappeared, specifically for group B patients. But we did not evaluate the shoulder external rotation and forward elevation because of poor results, so the external rotation needed to further improve by secondary procedures.

Elbow flexion is another important function. Both ICN transfer and FFGT could be used to reconstruct elbow flexion and achieve a good result. In general, FFGT reconstruction achieved better elbow flexion strength than ICN–to–musculocutaneous nerve transfer [31]. Moreover, the gracilis allows for earlier reinnervation and for the restoration of both elbow flexion and finger extension or finger flexion [32]. The SAN and ICN are typically used as donor nerves for the gracilis muscle [31, 33]. Our team usually used the SAN to reinnervate the first FFGT, and the ICN was used for the second FFGT. A functional elbow range of motion from 30° to 130°, or active flexion arc of 100°, is required to perform most activities of daily living [34]. In this study, 37 patients achieved elbow flexion recovery, the average elbow flexion degree was 107.2° (range 0–142°), and the average strength was 2.5 kg (range 0.5–5). These outcomes were superior to those in reports by Coulet [35].

Theoretically, this surgical method is expected to restore the pick-up function. However, the actual results were unsatisfactory; only four patients regained the pick-up function. The patients showed three gestures at the final follow-up, due to the imbalance in the power of the finger flexion CC7 transfer and finger extension gracilis. Most patients showed hyperextension of metacarpophalangeal joints, because the finger extension strength caused by gracilis was stronger than finger flexion strength, or no finger flexion recovery. This study also confirms that the outcome of pick-up function is not satisfactory, even if hand function is repaired early by CC7 transfer combined FFGT. We think that several factors affect the recovery of pick-up function such as the finger extension strength induced by gracilis was stronger than the finger flexion strength caused by CC7-LT, a long-distance nerve regeneration for the restoration of finger flexion, non-compliance with rehabilitation of patients, and the difficulty and complexity of cerebral cortical reorganization (neuroplasticity). Therefore, further surgery is needed to improve the finger flexion function, such as a second FFGT, tendon transfer, and MCP capsulodesis [36]. Our results are consistent with those reported in the literature that additional surgery can improve hand function and decrease the DASH score [37].

There were several limitations in this study. First, only thirty-nine patients took part in the follow-up research, resulting in a small sample size. Second, this study lacks preoperative DASH and NRS scores of these patients. In general, DASH and NRS scores after the surgery can decrease. Third, TBPA injuries have severe outcomes and lifelong disabilities. While we paid attention to the evaluation of the movement and sensory function of the affected limb, we ignored the assessment of psychology and ‘‘return to work’’.

## Conclusions

In summary, the “distal to proximal” reconstructive method for TBPA is an available method. This technique could help patients improve shoulder joint stability and the function of elbow flexion and finger extension and, more importantly, provide finger sensation and partial finger flexion function to the completely paralytic limb. However, in order to obtain a better pick-up function, patients need additional operations, such as wrist fusion, tendon transfer, and even secondary gracilis transplantation.

## Data Availability

All data gathered can be requested from the corresponding author.
